# Accuracy, repeatability, reproducibility and reference ranges of primary sclerosing cholangitis specific biomarkers from quantitative MRCP

**DOI:** 10.1007/s00261-025-04941-9

**Published:** 2025-04-17

**Authors:** Mukesh Harisinghani, Tom Davis, George Ralli, Carlos Ferreira, Bruno Paun, Andrea Borghetto, Andrea Dennis, Kartik Jhaveri, Filippo Del Grande, Sarah Finnegan, Michele Pansini

**Affiliations:** 1 Massachusetts General Hospital, Boston, USA; 2 Perspectum Ltd, Oxford, UK; 3 University of Toronto, Toronto, Canada; 4 Imaging Institute of Southern Switzerland (IIMSI), Ente Ospedaliero Cantonale, Lugano, Switzerland; 5 Oxford University Hospitals NHS Trust, Oxford, UK

## Abstract

**Purpose:**

To assess the repeatability and reproducibility of quantitative MRCP-derived metrics generated from MRCP + software, designed for assessing biliary tree health.

**Methods:**

Metric accuracy was assessed using a 3D-printed phantom containing 20 tubes with sinusoidally-varying diameters, simulating strictures and dilatations along ducts. Data from 80 participants (60 healthy volunteers and 20 with liver disease) was analysed in total. Repeatability and reproducibility of the quantitative metrics were assessed on Siemens, GE and Philips scanners at both 1.5T and 3T. All subjects were scanned on a Siemens Prisma 3T scanner which acted as the reference scanner. A subset of these participants also underwent scanning on the remaining scanners. Data from healthy volunteers was used to estimate the natural range of measured values (reference ranges). The reproducibility coefficient (RC) of 7 commonly reported quantitative metrics were compared between healthy controls and published values in primary sclerosing cholangitis (PSC) patients.

**Results:**

The phantom analysis confirmed measurement accuracy with absolute bias of 0.0-0.1 for strictures and 0.1–0.2 for dilatations across all scanners (95% limits of agreement within ± 1.0). In vivo, RCs for the quantitative MRCP-derived metrics across the scanners ranged from: 12.4–25.4 for total number of ducts; 4.9–7.9 for number of dilatations; 3.3–6.5 for number of strictures; 4.6–9.8 mm for total length of dilatations; 26.5–51.7 mm for total length of strictures; and 4.4–6.8 for number of ducts with a stricture or dilatation. Repeatability on the same scanner was generally better than comparisons across scanners. Six metrics demonstrated sufficient cross-scanner reproducibility to distinguish healthy volunteers from PSC patients.

**Conclusion:**

The precision of quantitative MRCP-derived metrics were sufficient to differentiate PSC and healthy subjects and should be well suited for multi-centre trials and assessment of biliary tree health.

**Supplementary Information:**

The online version contains supplementary material available at 10.1007/s00261-025-04941-9.

## Introduction

Primary sclerosing cholangitis (PSC) is a chronic liver disease, characterised by multi-focal strictures throughout the biliary tree [1]. Currently, the only definite treatment for PSC is liver transplant. PSC patients live with ongoing risk of cholangitis and liver failure. As such, they are carefully monitored for signs of disease progression and risk of serious clinical events [2–4]. Patients showing signs of progression may be eligible for a liver transplant, the prognosis of which is generally improved the earlier the patient is identified as needing a transplant. In early stages of the disease, and in the presence of overlap features with diseases such as autoimmune hepatitis (AIH), PSC can be challenging to both diagnose [5] and to monitor for signs of progression.

Imaging of the biliary tree with cholangiopancreatography (CP) plays a vital role in the assessment and monitoring of PSC cases. The main types of CP are magnetic resonance (MRCP) and endoscopic retrograde (ERCP). ERCP is invasive and poses significant risks for patients, carrying a 0.2-1% risk of mortality and a 9.8–15.9% risk of complications [6]. Thus, MRCP is commonly used as a non-invasive alternative. However, MRCP evaluations are subjective and depend heavily on radiologist experience and image quality, resulting in high levels of inter- and intra-observer variation [7, 8]. For example, the MRCP-derived ANALI score was found in one study to have an inter-observer variability of over 80% [8].

To overcome these limitations and standardize use of MRCP for the management of PSC patients, quantitative analysis tools have been developed [9] and investigated [10–19]. One such tool is MRCP+, a post-processing software that produces a 3D rendering of the biliary tree and a series of quantitative metrics of duct morphology. These tools are used to post-process the MRCP images, producing a 3D rendering of the biliary tree and a series of quantitative metrics of duct morphology. While these measurements aim to provide objective assessment of biliary structures, their clinical value depends on reliability and consistency. In fact, as with any image processing tools, measurements derived from MRI data are subject to noise, both due to random measurement variation and differences between MRI scanners, so reporting on their accuracy and precision is essential. Accuracy can be assessed by comparing the measured metrics to a known ground-truth, which is often done using manufactured phantoms. Precision is commonly assessed by measuring the repeatability and reproducibility. The former is defined as agreement between measurements under similar conditions (e.g. repeated measurement on a single scanner) and the latter as agreement under different conditions (e.g. different manufacturers and field strengths). An early version of MRCP+, which focused only on measuring the width of individual ducts and the biliary tree volume, was shown to have excellent cross-scanner reproducibility and sub-millimetre accuracy using 1.1 mm isotropic resolution 3D-MRCP images [9].

In PSC, strictures and dilatations are key diagnostic and prognostic features. More recent studies using quantitative MRCP analysis have focused on metrics such as the numbers of strictures and dilatations and measurements of their lengths and severities. Several studies have demonstrated the utility of these metrics for patient stratification [13–16], disease monitoring [15] and predicting clinical outcomes in PSC [17–19]. Despite these promising results, the precision of these newer metrics has not yet been reported. Furthermore, ranges for these metrics, calculated in a healthy population (reference ranges), are needed to confidently distinguish patients with potential disease from the natural variation observed in the healthy population and thereby facilitate clinical adoption.

Here we report on the accuracy and precision of advanced quantitative MRCP metrics derived from MRCP + software, with a particular focus on those metrics most reported from the literature and perceived physician utility. Repeatability and reproducibility are measured using human volunteers, while accuracy is established using a purpose-built phantom. A population of subjects with no known liver disease is also used to establish the healthy reference ranges for each metric studied.

## Methods

### Study design and participants

For repeatability and reproducibility scans the study was reviewed by the South Central– Oxford C Research Ethics Committee (REC reference: 17/SC/0459) and written informed consent was obtained from all participants. Data from a further 20 healthy volunteers scanned once on Siemens 3T as part of a separate study (REC reference: 18/SC/0367), were used in the calculation of reference ranges. This additional data was not used in the measurement of repeatability and reproducibility.

**Fig. 1:**
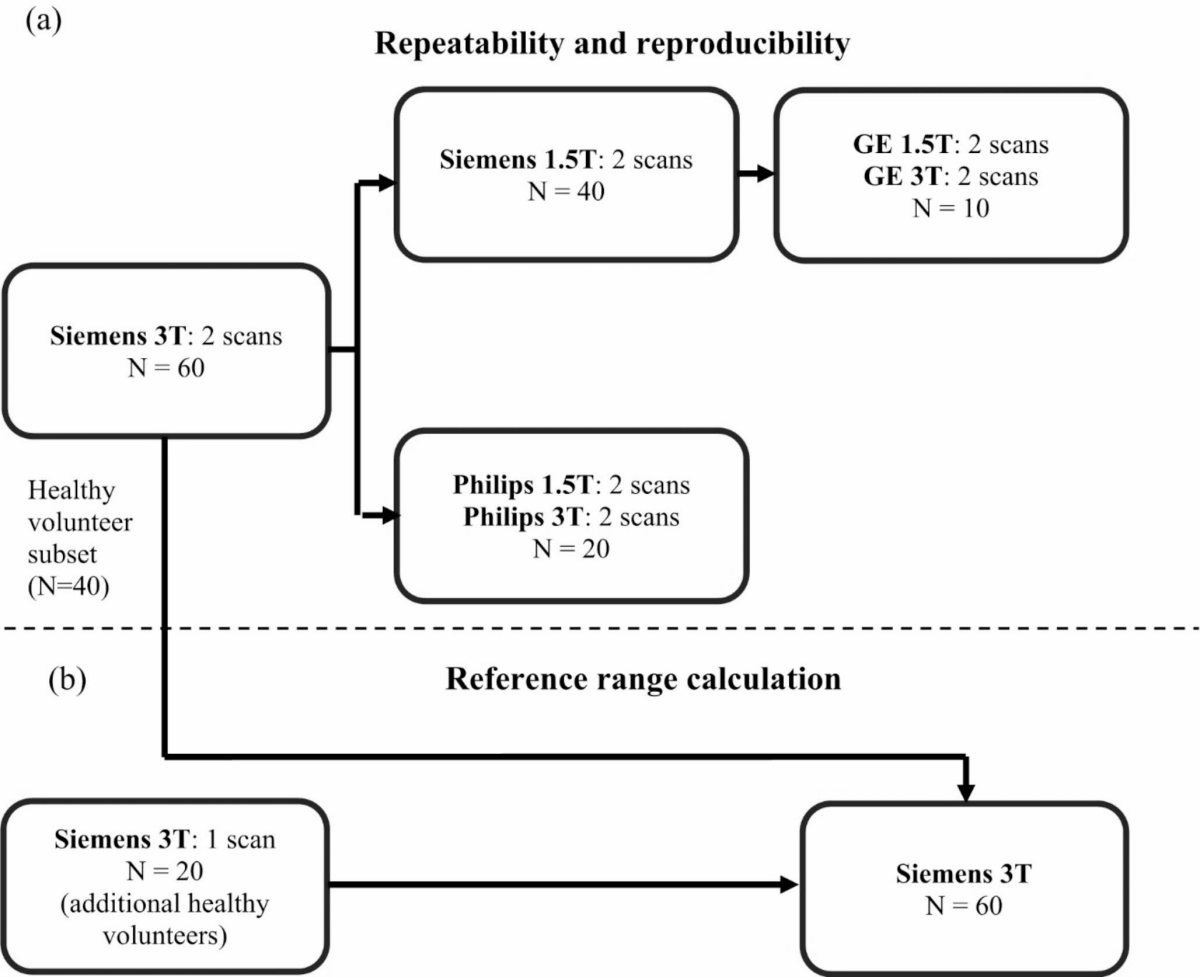
Flowcharts summarising the distribution of human subjects for (**a**) the repeatability and reproducibility experiments and (**b**) the reference range calculation

Data was collected from 80 participants,60 healthy volunteers, 10 with parenchymal liver disease and 10 with biliary disease, including primary sclerosing cholangitis (PSC), primary biliary cholangitis and gallstones. All participants were required to maintain nil per os for 4 h before the MRI examination. Of the 80 participants, 60 were used to determine the repeatability and reproducibility of quantitative metrics calculated using the MRCP + software package (version 2, Perspectum Ltd., Oxford, UK). All 60 were scanned on a Siemens Prisma 3T scanner (Siemens Healthineers, Erlangen, Germany). Subsets of these participants were also scanned on a Siemens AvantoFit 1.5T; a GE Optima 450w 1.5T and GE Discovery 3T (GE Healthcare, Milwaukee, WI); and a Philips Ingenia 1.5T and Ingenia 3T (Philips Healthcare, Best, Netherlands). Figure 1 illustrates how the patients were distributed between the scanners. In each scanning session patients were scanned twice with the same sequences, with patients exiting and re-entering the scanner. The remaining 20 participants were only scanned once on the Siemens 3T and used as part of the reference range analysis.

Repeatability was assessed for each scanner by comparing the results from the two repeat scans. Reproducibility was determined by comparing the results from the first repeat scan on each scanner to those obtained from the same patients on the Siemens 3T, which was defined as the reference scanner as this scanner had the greatest number of datasets. The study design is summarised in Fig. 1, and participant demographics are shown in Table 1.

**Table 1:** Demographics of All recruited participants within the repeatability and reproducibility testing. Note that because several patients are scanned on multiple scanners (e.g. All 10 GE patients were also scanned on Siemens 1.5T), the number of diseased patients in each scanner May not sum up to the total number on Siemens 3T. Figure 1 shows how the patients are distributed between scanners. BMI– Body mass index, PSC - Primary sclerosing cholangitis, PBC - Primary biliary cholangitis, HCV - Hepatitis C virus, NAFLD– Non-alcoholic fatty liver disease

	Scanning groups				Reference Ranges
Scanner	Siemens 3T	Siemens 1.5T	GE (1.5 & 3T)	Philips (1.5 & 3T)	Siemens 3T
N	60	40	10	20	60
Male/Female	28/32	22/18	3/7	10/10	30/30
Age (years)	39.3	45.9	28.8	40.7	34.4
BMI (kg/m2)	25.5	26.1	24.9	24.2	24.0
**Reported health conditions**					
PSC	6	6	0	0	0
PBC	5	4	0	1	0
HCV	1	1	0	0	0
NAFLD	6	5	0	0	0
HC	2	2	0	0	0
Veno-occlusive disease	1	1	1	0	0
Liver Cysts	2	0	0	2	0
Gallstones	1	0	0	1	0
**Ethnicity**					
African	2	N/A	N/A	2	N/A
Chinese	3	N/A	N/A	3	N/A
Indian	1	N/A	N/A	1	N/A
White British	7	N/A	N/A	7	N/A
Other white	7	N/A	N/A	7	N/A
Not reported	40	40	10	0	60

### MRI acquisition

**Fig. 2:**
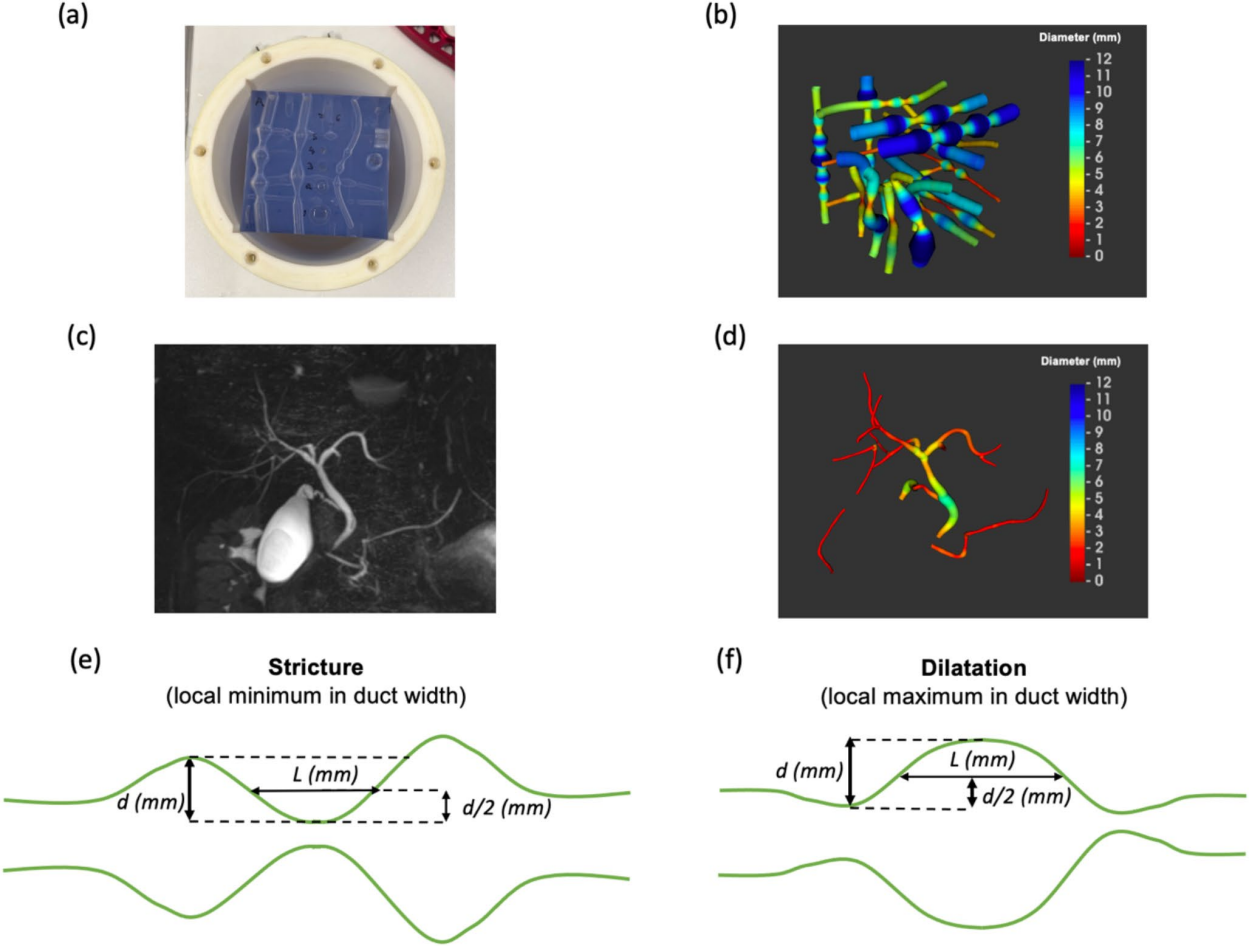
(**a**) Photograph of the 3D-printed phantom placed inside outer cylinder for flood-filling. (**b**) MRCP + model of the phantom, derived from a GE 1.5T scan, with tubes color-coded by diameter. (**c**) Maximum intensity projection MRCP image of patient scanned on Siemens 3T and (**d**) corresponding MRCP + model. Schematic diagrams illustrating a stricture (**e**) and dilatation (**f**), which are defined as local minima and maxima in the duct width profile, respectively, whose severity d (the width change from the neighbouring extremum with the closest width) is greater than both 1 mm and 30% and length L is the distance between the two points on either side at which the duct width has changed by d/2

MRCP images were acquired using a 3D multi-shot fast/turbo spin echo sequence to generate heavily T2-weighted volumetric images, highlighting slow-moving fluids such as bile. Each image was acquired with an isotropic voxel resolution of 1.1 × 1.1 × 1.1 mm. Respiratory gating was performed using navigator tracking, with data acquired at the expiration phase of the breathing cycle. Acquisition parameters are shown in supplementary table S1. Figure 2 shows an example MRCP acquisition on Siemens 3T.

### Manufactured phantom

Accuracy was assessed using a custom-designed 3D-printed phantom. The phantom is a block of TuskXC2700T material containing 20 hollow tubes with undulating widths, designed to simulate strictures and dilatations of varying severities and lengths. The expected values of the MRCP + metrics were calculated for each tube using the dimensions in the design specification and are shown in supplementary table S3. The phantom was flood-filled with water doped with 2.4 mM nickel chloride and 2.0 mM sodium benzoate per litre and scanned using the same scanner types and sequences as the human volunteers. Figure 2 (a) and (b) show a photograph of the phantom and renderings of the tubes, respectively. Further details of technical specifications can be found within supplementary materials.

### Quantitative image analysis

MRCP images were processed using MRCP+ (Version 2, Perspectum Ltd, Oxford, UK), which has also been used in several studies evaluating the utility of quantitative MRCP metrics in PSC patients [13–19]. For completeness, we briefly outline the key processing steps: the software first applies a tubular enhancement algorithm [21] to the images, and a threshold is automatically computed within a region of interest centred on the branching point of the common bile duct. 3D tubular objects whose intensity lies above this threshold are rendered, and the user segments the biliary tree by selecting regions corresponding to pancreatobiliary structures. MRCP + then detects the centrelines of the selected ducts, computes point-wise diameter measurements and reconstructs a 3D model of the biliary tree, color-coded by diameter. Figure 2 (b, d) shows example MRCP + models, and [9, 21] provides a more technical overview of the underlying algorithms.

To detect strictures and dilatations, the software detects local maxima and minima along the duct width profiles (Fig. 2 (e, f)). The absolute and percentage difference in width between each extremum and the neighbouring extremum with the closest width, respectively termed the absolute and relative severity, are computed. Strictures and dilatations are defined as local minima and maxima, respectively, with an absolute severity of ≥ 1 mm and a relative severity of ≥ 30%. Figure 2 (e, f) illustrate how strictures and dilatations, along with their length and severity, are calculated.

The number, length and severity of the strictures and dilatations are computed for each modelled duct. The 3D-printed phantom was used to evaluate the accuracy of these per-duct metrics. The software also provides summary metrics for the entire biliary tree. Each metric is defined in supplementary table S2, and the precision (repeatability/reproducibility) of these whole-tree metrics was evaluated using the in vivo subjects.

All cases were processed by a single operator. To test intra- and inter-operator variability, a subset of 40 S 3T cases were re-processed by this operator and were compared to a second operator. Both operators were radiographers (17 and 5 years of experience for the primary and secondary operators, respectively) who were familiar with hepatobiliary anatomy and pathologies, and had received formal training in MRCP + software operation from the developer.

### Statistical analysis

Bland-Altman analysis was used to compare repeated sets of measurements. This involves calculating the bias (defined as the mean difference between the repeated measurements), and the 95% limits of agreement (LoA) (1.96 times the standard deviation). The reproducibility coefficient (RC), defined as 2.77 times the within-subject standard deviation, was also calculated for the in vivo data.

For the reference range calculations in the 60 healthy volunteers scanned on Siemens 3T, lower and upper thresholds of the for each metric were calculated using mean ± 1.96×standard deviation for metrics that were normally distributed and the 2.5th and 97.5th percentiles for those which were not. All analyses were performed in R (version 4.3 or later, R Project for Statistical Computing, Vienna, Austria).

## Results

Due to the large number of metrics produced by MRCP+, here we focus on the seven metrics that are most frequently reported in the literature as having potential clinical utility in PSC [13–19]. These are: (1) the total number of ducts; (2) the total number of ducts with stricture or dilatation; (3) the number of dilatations and (4) total length of dilatations; (5) the number of strictures and (6) total length of strictures; and (7) the percentage of ducts with a diameter of 3–5 mm. The results for the remaining metrics are shown in the supplementary material.

### Participant characteristics

Sixty of the 80 participants were included in the repeatability and reproducibility analysis, 32 of whom were female, with a mean age of 39 (SD 14). 40 of these participants had no known diagnosis of liver or biliary disease. Participants scanned on the GE scanner were significantly younger (mean age 28 years) and predominantly free of any known liver disease diagnosis. Participant demographics are summarised in Table 1.

### 3D-printed phantom accuracy

Metric accuracy are shown in Table 2. As each tube is treated separately, this table shows the results for the per-tube single duct metrics that are used to calculate the whole tree metrics reported for the in vivo results. Strictures and dilatations were detected with high accuracy, with the limits of agreement on the numbers of strictures and dilatations being ≤ 1.0 across all scanners. The accuracy of all single-duct MRCP+ metrics is shown in supplementary table S5.

**Table 2:** Accuracy of the single duct metrics, comparing the per-tube Phantom metrics to the expected values. Results are reported as the bias, with the square brackets showing the lower and upper 95% limits of agreement

Metric	Siemens 1.5T	Siemens 3T	GE 1.5T	GE 3T	Phillips 1.5T	Phillips 3T
Total number of dilatations	0.1 [-0.4,0.5]	0.2 [-0.6, 1.0]	0.1 [-0.4, 0.5]	-0.1 [-0.6, 0.5]	-0.1 [-0.5, 0.4]	0.0 [-1.0, 1.0]
Total number of strictures	0.1 [-0.4-0.5]	0.1 [-0.4, 0.5]	0.0 [0.0, 0.0]	0.1 [-0.9, 1.0]	0.1 [-0.5, 0.7]	0.0 [0.0, 0.0]
Stricture length sum (mm)	0.4 [-3.3, 4.1]	0.1 [-4.0, 4.2]	0.3 [-1.9, 2.5]	1.0 [-6.9,8.9]	-0.3 [-3.5, 2.9]	0.0 [-1.9, 1.8]
Dilatation length sum (mm)	0.9 [9.0, 10.9]	1.7 [-8.3, 11.6]	1.6 [-9.9, 13.1]	0.5 [-13.8, 14.8]	2.2 [-8.6, 12.9]	0.6 [-16.1, 17.4]

### In vivo repeatability and reproducibility

Tables 3 and 4 show the in vivo test-retest repeatability (on each scanner) and cross-scanner reproducibility vs. Siemens 3T, respectively, of the whole-tree MRCP + metrics. The cross-scanner reproducibility coefficients are generally slightly higher than test-retest ones. Results for the inter- and intra-operator repeatability are shown in supplementary table S4.

**Table 3:** Repeatability of PSC specific metrics across all scanners examined. LoA– Limits of agreement; RC– Repeatability coefficient

Metric	GE 1.5T		GE 3T		Siemens 1.5T		Siemens 3T		Phillips 1.5T		Phillips 3T	
	LoA	RC	LoA	RC	LoA	RC	LoA	RC	LoA	RC	LoA	RC
Total number of ducts	[-12.6, 17.2]	14.8	[-13.5, 12.2]	12.2	[-25.6, 21]	23.5	[-9, 11.3]	10.3	[-15.3, 10.9]	13.4	[-11.1, 10.2]	10.3
Total number of dilatations	[-5.2, 6.8]	5.9	[-5.2, 5]	4.8	[-4.7, 6.5]	5.8	[-4.1, 4.7]	4.4	[-4.8, 4.7]	4.6	[-3, 2.4]	2.7
Dilatation length sum (mm)	[-4.5, 6.3]	5.5	[-6.9, 7.2]	6.7	[-5.7, 7.7]	6.9	[-5.4, 7.6]	6.8	[-5.9, 6.3]	6	[-4.7, 4]	4.2
Total number of strictures	[-2.9, 4.5]	3.8	[-4.2, 4]	3.9	[-4.4, 5.9]	5.3	[-3.7, 5.1]	4.6	[-4, 4.9]	4.4	[-3.3, 3]	3.1
Stricture length sum (mm)	[-25.9, 41.9]	35.8	[-29, 13.2]	25.2	[-37.3, 53.3]	47.4	[-41.1, 55.1]	49.6	[-48.9, 52.1]	49.1	[-25.4, 20.9]	22.7
Total number of ducts with a stricture or dilatation	[-5.2, 6.2]	5.5	[-5.5, 5]	5	[-4.9, 6.5]	5.8	[-3.9, 5.2]	4.7	[-3.9, 3.8]	3.8	[-2.5, 1.6]	2.2
Percentage of ducts with diameter 3–5 mm*	[-0.4, 0.2]	0.3	[-0.1, 0.1]	0.1	[-0.3, 0.3]	0.3	[-0.2, 0.1]	0.2	[-0.1, 0.2]	0.2	[-0.2, 0.2]	0.2

*Percentage expressed as a fraction (i.e. divided by 100)

**Table 4:** Reproducibility of PSC specific metrics across all scanners examined versus the reference scanner (Siemens Prisma 3T)

Metric	GE 1.5T		GE 3T		Siemens 1.5T		Phillips 1.5T		Phillips 3T	
	LoA	RC	LoA	RC	LoA	RC	LoA	RC	LoA	RC
Total number of ducts	[-22.1, 20.7]	20.4	[-13.4, 14.6]	13.4	[-19.6, 28.7]	25.4	[-12.1, 16.7]	14.7	[-8.5, 14.3]	12.4
Total number of dilatations	[-7.9, 4.9]	6.7	[-4.6, 5.6]	4.9	[-7.2, 5.3]	6.4	[-7.8, 8.3]	7.9	[-6.1, 7.3]	6.6
Dilatation length sum (mm)	[-5.9, 6.6]	6	[-6.8, 8.6]	7.5	[-9.2, 10.5]	9.8	[-4.7, 4.7]	4.6	[-4, 5.3]	4.7
Total number of strictures	[-4.2, 4.4]	4.1	[-3.8, 5]	4.3	[-6.4, 6.8]	6.5	[-3.8, 3.4]	3.5	[-2.7, 3.7]	3.3
Stricture length sum (mm)	[-55.4, 53.2]	51.6	[-32, 42.7]	37	[-49.1, 55]	51.7	[-26.5, 27.8]	26.5	[-29.3, 42.7]	37.4
Total number of ducts with a stricture or dilatation	[-5, 4.2]	4.5	[-2.7, 5.1]	4.4	[-7.3, 6.5]	6.8	[-5.8, 6.4]	6	[-4.3, 6.4]	5.6
Percentage of ducts with diameter 3–5 mm*	[-0.2, 0.3]	0.3	[-0.3, 0.2]	0.2	[-0.3, 0.2]	0.3	[-0.2, 0.3]	0.3	[-0.3, 0.3]	0.3

LoA– Limits of Agreement; RC– Reproducibility Coefficient. *Percentage expressed as a fraction (i.e. divided by 100)

**Fig. 3:**
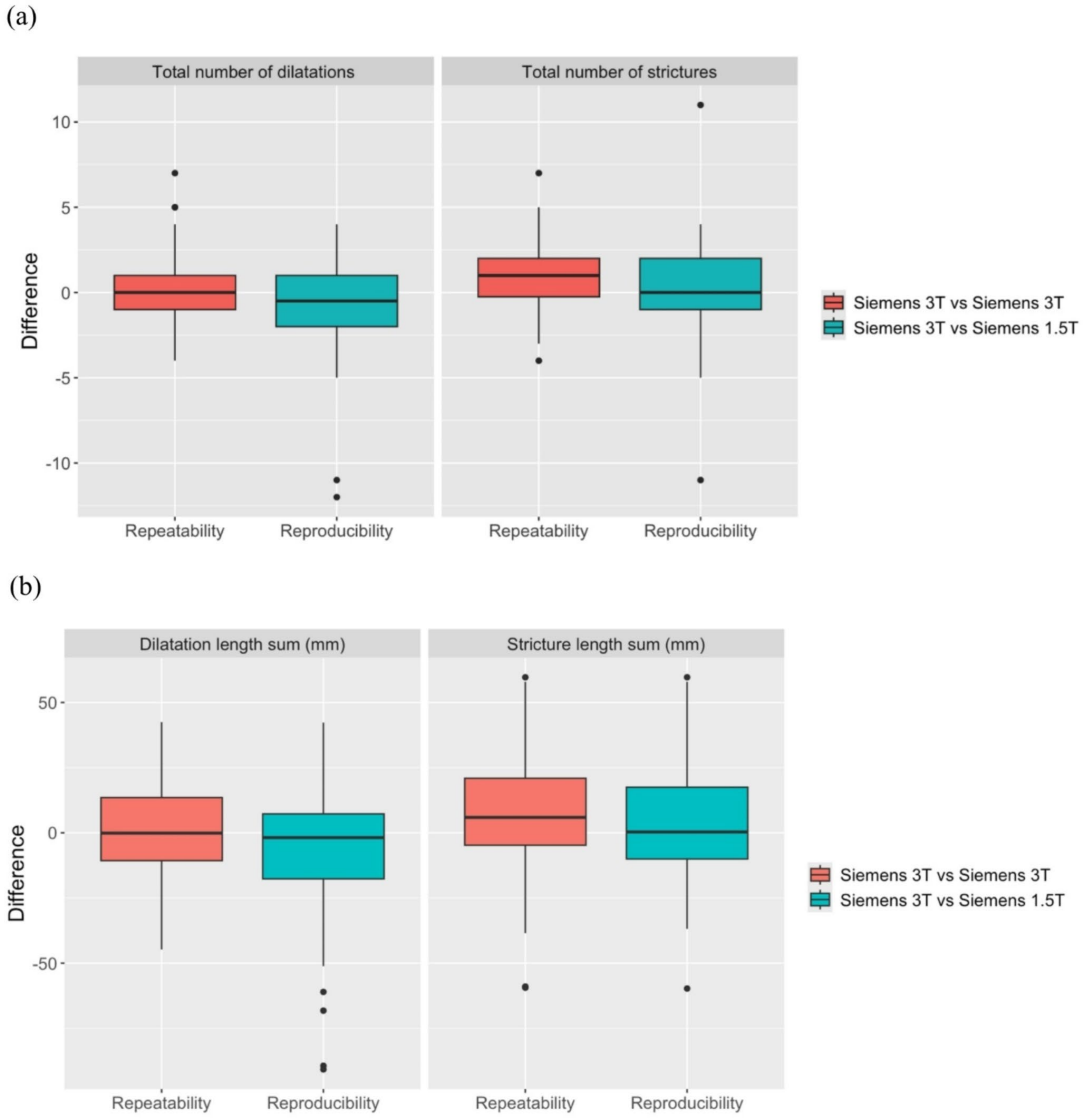
Boxplots illustrating the repeatability (on Siemens 3T) and reproducibility (Siemens 3T vs. Siemens 1.5T) of the number (**a**) and lengths (**b**) of strictures and dilatations. The values plotted are the differences in metric values between the two scans. Results are shown for the Siemens group as these had the largest number of patients with liver disease as well as healthy volunteers, and consequently the highest range of metric values

To illustrate repeatability and reproducibility, Fig. 3 shows boxplots comparing differences in the number and lengths of strictures and dilatations between repeated Siemens 3T scans (repeatability) and between Siemens 1.5T and 3T scanners (reproducibility). Additional metrics (total number of ducts, total number of ducts with a stricture or dilatation and percentage of ducts with diameter 3–5 mm) are shown in supplementary figure S1.

### Healthy reference ranges

Table 5 shows the metric reference ranges calculated for the 60 healthy volunteers scanned on Siemens 3T, none of whom had any known previous diagnosis of biliary disease. To contextualise these values, the same metrics from 77 PSC cases reported by Cazzagon et al. [19] are also shown. This PSC population contains patients with a range of disease severities: ANALI scores ranged from 0 to 5 without and 0–2 with gadolinium; 24 patients had intrahepatic PSC only and 53 had intra- and extrahepatic PSC– see Table 1 in referenced publication [19] for full demographic details.

**Table 5:** Median, IQR, and reference ranges for PSC-specific metrics from a cohort of healthy participant. For further contextualisation, metric values in 77 PSC cases previously reported in [19] are shown for comparison

	Healthy subjects (*N* = 60)		PSC subjects reported in [19] (*N* = 77)
**Measurement**	**Median (IQR)**	**Reference range**	**Median (IQR)**
Total number of ducts	20 (10.5)	0–38	86 (84)
Total number of dilatations	3 (2.5)	0–8	21 (23)
Dilatation length sum (mm)	33.8 (27.1)	0–76	123.5 (139.2)
Total number of strictures	2 (2.0)	0–6	11 (11)
Stricture length sum (mm)	20.7 (18.6)	0–59	77.6 (106.8)
Total number of ducts with a stricture or dilatation	4 (3.5)	1–9	20 (21.5)
Percentage of ducts with diameter 3–5 mm (%)	20 (11)	0–38	22 (15)

## Discussion

The aim of this study was to report the accuracy and precision of quantitative metrics of the biliary tree, measured from non-invasive MRCP images using commercially available software, which has received increasing attention for the assessment and monitoring of patients with PSC. The detection and measurement of strictures and dilatations had excellent accuracy and negligible bias across all the scanners tested, as demonstrated by the results on phantoms with known values. The In vivo precision was generally better for test-retest repeatability than cross-scanner reproducibility. However, for 6/7 metrics both test-retest and cross-scanner reproducibility coefficients, which capture measurement error were lower than the median differences observed between healthy volunteers and published PSC values. This suggests that the metrics are sufficiently precise to distinguish these populations.

**Fig. 4:**
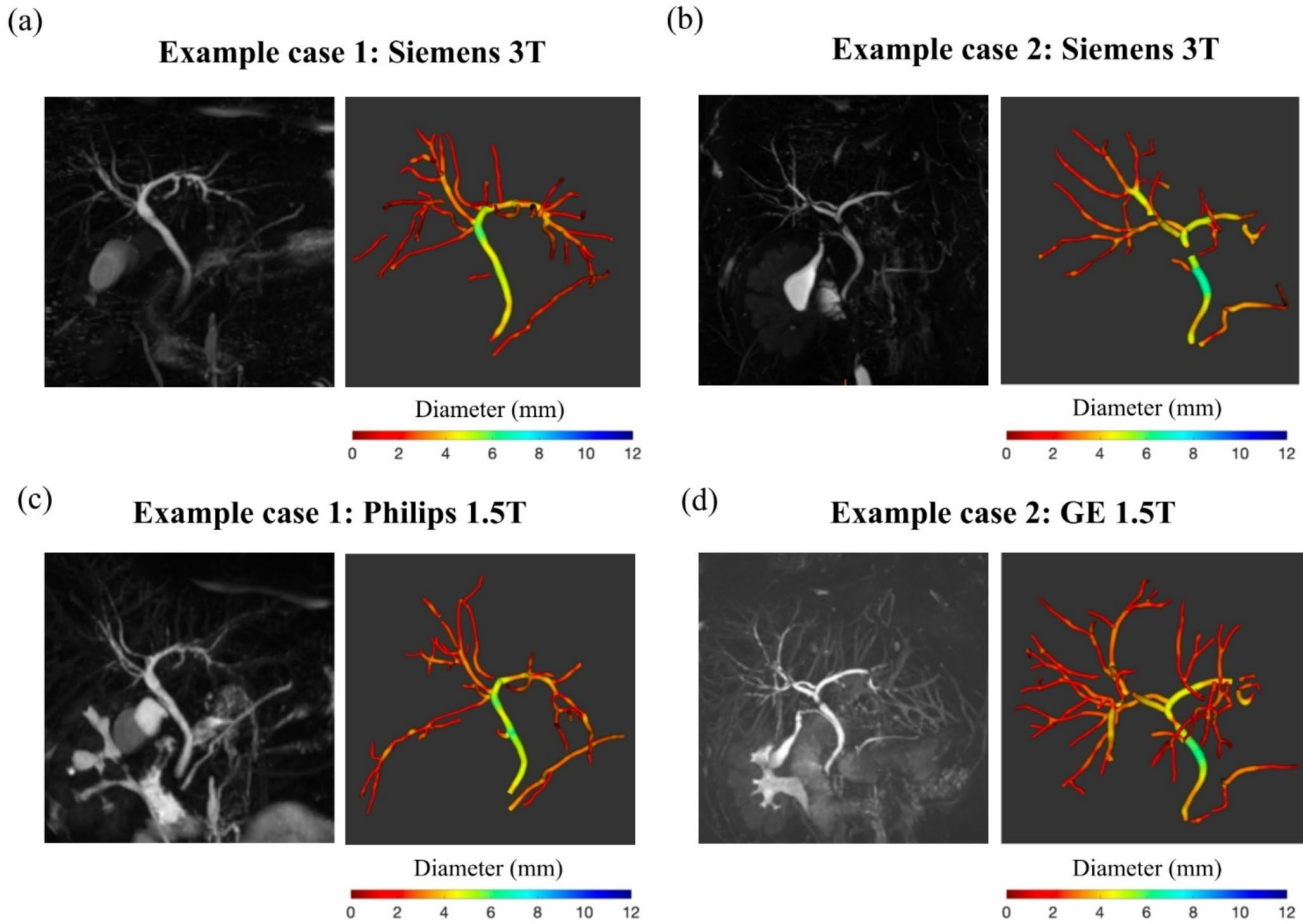
Examples of challenging pairs of acquisitions with reduced cross-scanner consistency. (**a**) and (**b**) show MRCP MIPs of two separate subjects (cases 1 and 2, respectively), acquired on Siemens 3T, alongside their MRCP + models. The corresponding results shown for case 1 (**c**) and case 2 (**d**) were acquired on Philips 1.5T and GE 1.5T, respectively. Both 1.5T images have worse background suppression, making analysis more challenging. For case 2, more faint vessel-like structures are visible in the 1.5T scan (**d**), leading to more ducts being modelled than for the corresponding 3T scan (**b**)

Reproducibility of MRCP + metrics was assessed across scanners with varying field strengths (1.5T and 3T), and while precision was generally sufficient to distinguish PSC and healthy populations, some differences were observed. Metrics such as the total number of strictures and dilatations demonstrated slightly better reproducibility at 3T. However, certain metrics, including the total number of ducts with stricture or dilatation, showed consistent reproducibility across field strengths, reflecting their robustness to scanner variations. It was observed during the analysis that the 3T MRCP acquisitions often achieved superior background suppression, making the selection and modelling of the ducts easier, which may partly explain the slightly improved reproducibility of some metrics at 3T. Figure 4 presents selected case examples demonstrating varying levels of repeatability between scans acquired at 1.5T and 3T, which also illustrate the superior background suppression at 3T. These results indicate that while field strength can influence precision for some metrics, others remain stable across different imaging environments, supporting their suitability for multi-center trials. Differences in sequence parameters, including echo times, may also contribute to the observed variability, underlining the importance of harmonizing imaging protocols when incorporating quantitative MRCP metrics into clinical workflows.

The tighter limits of agreement for the phantom accuracy compared with that of the in vivo precision data is likely because the former is unaffected by issues such as patient motion and bright gastrointestinal structures located near the biliary tree if the patient has not fasted prior to the scan. This suggests that patient compliance with breathing and fasting instructions may be a dominant source of error in the quantitative metrics compared to error caused by the underlying acquisitions or image processing algorithms.

For each in vivo measure of metric consistency reported here, further contextualisation is needed to assess whether these values are ‘good’. The interpretation of the RC is that, in 95% of cases, noise induced by repeated measurements will not exceed this value, thus a change greater than the RC would indicate a true change. One way to contextualise the RC values is to compare them to the differences typically seen in healthy and PSC populations, as shown in Table 5. For example, the median (IQR) number of strictures in the PSC and healthy groups are 11 (11) and 2 (2), respectively. If a treatment was expected to reduce the number of strictures to the value seen in a healthy population, the change would require a reduction in at least 9 strictures. Any change of this magnitude would be greater than the range of RC values (3.1–6.5), (Tables 3 and 4), and therefore detectable on an individual level. By contrast, the difference in equivalent values for the percentage of ducts with diameter 3–5 mm: 22 (15) vs. 20 (11) for the PSC and healthy groups, respectively, is less than the range of RC values (10–30), suggesting this measure may be insensitive to differences between PSC and healthy patients. This is consistent with the findings of Trivedi et al. [15], where no significant difference in this metric between PSC and healthy populations were reported. However, while not diagnostic, this metric has shown promise as a prognostic marker for PSC [18, 19], suggesting that it may capture aspects of PSC relevant to later stage disease.

The MRCP + software has a relatively low threshold for detection of strictures (absolute change in width of 1 mm), which makes it sensitive to subtle changes in duct width that may not be recorded by a radiologist. This explains why the healthy reference range for number of strictures (as defined by MRCP+) is 0–6, while radiologists, who typically focus on severe duct stenosis, may report fewer or no strictures in healthy subjects. While the exact definition of strictures used by the software may lead to more strictures being detected in healthy biliary trees than may be reported by a radiologist, strictures measured according to this definition have nevertheless been found to have good utility for diagnosing and monitoring PSC cases [13–19]. In fact, the ability to detect subtle biliary structures through quantitative analysis may characterise diffuse patterns and explain why this approach has previously outperformed other traditional assessments [18, 19].

Conventional MRCP image interpretation is subjective, with many studies reporting poor inter-observer variability. For example, Selvaraj et al. [13] reported that even simple metrics of maximum duct diameter had poor inter-observer agreement when measured manually by expert radiologists (ICC = 0.40 for left hepatic duct). Furthermore, Grigoriadis et al. [8] found that the semi-quantitative assessment of more complex features, including dilatations, using the ANALI score had a Cohen’s kappa score of 0.38, while strictures characterised by the DiStrict score depended heavily upon radiologist experience, with less experienced radiologists demonstrating poor agreement (ICC 0.48; 95% CI 0.05–0.72) [7].

Therefore, the performance demonstrated by the quantitative MRCP metrics highlights the improvements possible by converting to a fully quantitative and objective assessment of MRCP examinations. This is particularly important when considering that standardizing assessments of biliary health could ensure patients receive the most appropriate care in a timely fashion. An objective assessment is also imperative in a drug development setting, where quantitative, reliable metrics could detect subtle changes in biliary tree health. In multi-centre trials with longitudinal assessments of disease, an understanding of the magnitude of change due to measurement noise is required. Future work should focus on estimating the clinically meaningful change in quantitative MRCP metrics, to strengthen the utility as clinical biomarkers and as objective endpoints in PSC clinical trials.

Due to practical constraints, it was not possible to scan all subjects on all scanners, which were located in different cities. Furthermore, the GE and Philips groups had far fewer subjects with biliary or other liver diseases, and thus the range of metric values was lower in this group. Nevertheless, the worst-case RC values across all scanners still enabled differentiation of healthy and PSC cases. Furthermore, the metric accuracy assessed using the phantom– which provided a true gold-standard and covered a broad range of metric values– was similar across all scanners.

While this study demonstrates promising results for quantitative MRCP metrics, practical challenges may affect their implementation in AI-driven clinical workflows. Our study population had a mean age of 39 years and mean BMI of 25.5 kg/m², which may not fully represent the diversity of patients encountered in clinical practice. When integrating these metrics into automated AI systems, variations in image quality due to patient factors (respiratory motion, body habitus) could impact performance. In practice, published studies of patients including those with PSC, PBC or AIH have shown successful return of MRCP + reports in 90–95% of prospectively collected MRCP scans [13, 15].

Several steps are already in place as part of the MRCP + workflow which address potential sources of variability introduced more generally within AI-driven workflows:

(1) MRCP + checks the DICOM files when loading to ensure the acquisition parameters are within acceptable ranges. (2) Operators are trained to recognize and reject cases affected by severe artefacts. (3) MRCP + uses a standardized MR-protocol where possible, which has been optimised for image quality and robustness. As new AI-enhanced and/or accelerated MR-imaging techniques become commonplace, underlying MRCP data quality may also be improved, reducing potential impact of motion artefacts. Within fully automated AI-workflows, step (2) above would likely be automated, although this would require significant training data to justify removing the security of the human-in-the-loop checkpoint.

## Conclusion

The results of this study suggest that certain quantitative MRCP analysis produces reliable metrics suitable for multi-centre trials and longitudinal assessment of biliary tree health. This provides further support for the potential of quantitative MRCP metrics as clinical biomarkers and as objective endpoints in PSC clinical trials. The demonstrated reproducibility of these metrics across different scanners and field strengths ensures their suitability for integration into AI-driven diagnostic workflows, which could enable automated and standardized disease assessment that supports clinical decisions-making and patient care.

## Electronic supplementary material

Below is the link to the electronic supplementary material.

Supplementary Material 1

## Data Availability

Data Availability Statement: the datasets generated and analysed during the current study are not publicly available due to privacy restrictions relating to human participant data but are available from the corresponding author upon reasonable request and with appropriate ethical approvals.
