# Supplementary material for: Accuracy, repeatability, reproducibility and reference ranges of primary sclerosing cholangitis specific biomarkers from quantitative MRCP
Source: Abdom Radiol (NY). 2025 Apr 17;50(11):5211–22. doi: 10.1007/s00261-025-04941-9 (PMC12568806; doi:10.1007/s00261-025-04941-9)
Supplement: Supplementary file 1 — Supplementary Material 1 [file 261_2025_4941_MOESM1_ESM.docx]

Figure S1. Boxplots illustrating the repeatability (on Siemens 3T) and reproducibility (Siemens 3T vs Siemens 1.5T) of the total number of ducts (a), the number of ducts with a stricture or dilatation (b), the percentage of ducts with diameter 3-5 mm (c). The values plotted are the differences in metric values between the two scans. Results are shown for the Siemens group as these had the largest number of patients with liver disease as well as healthy volunteers, and consequently the highest range of metric values.


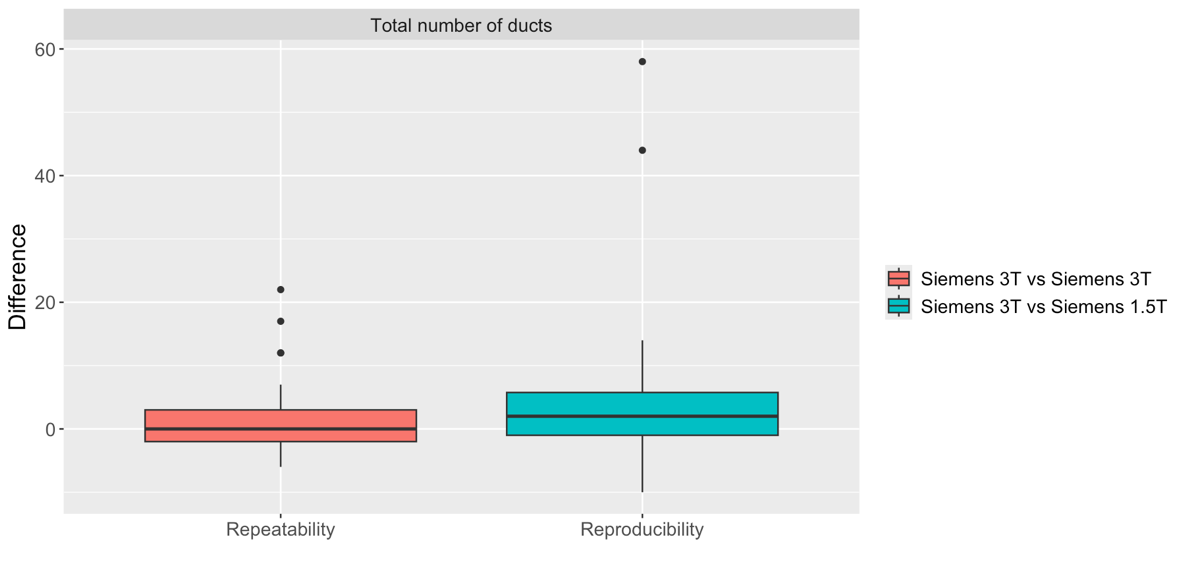

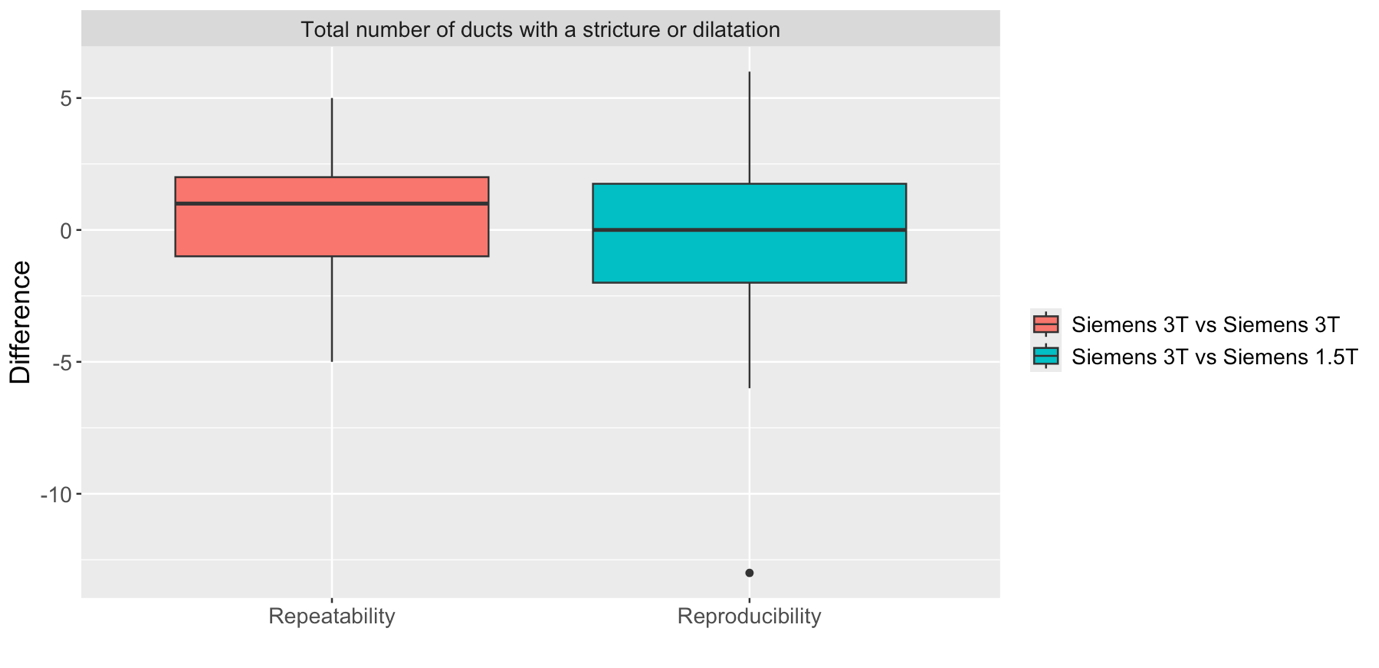

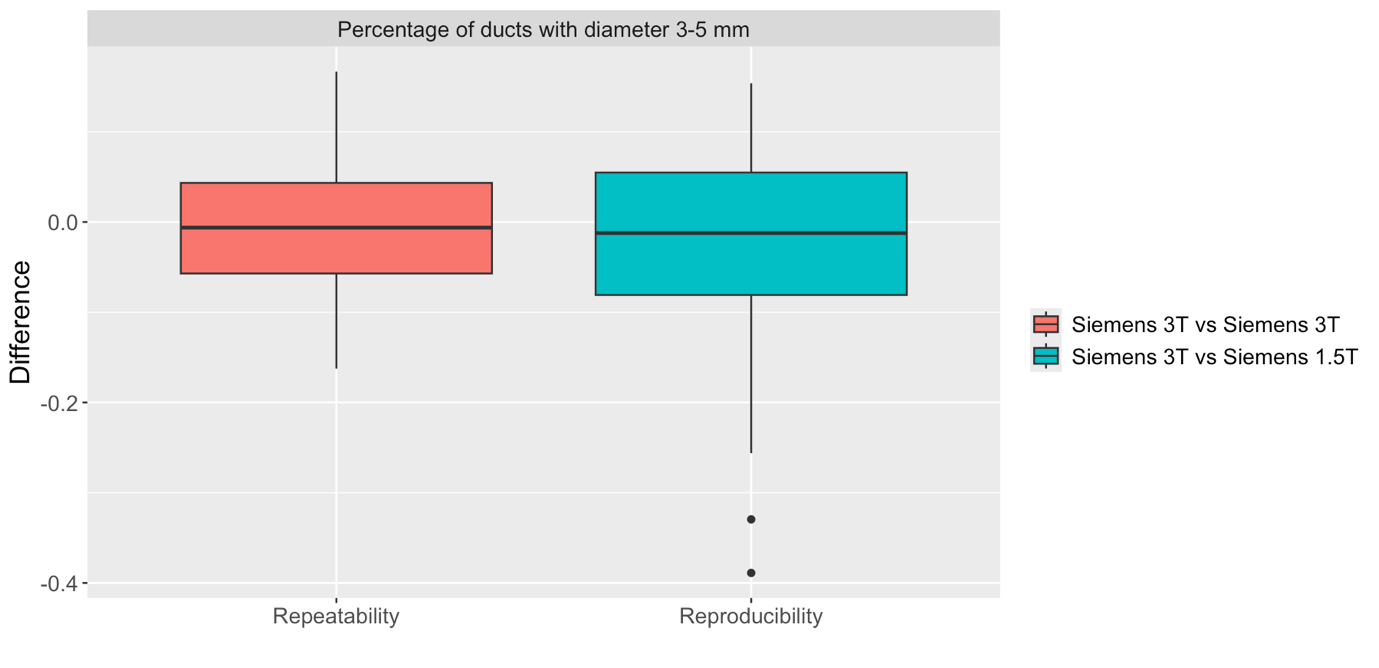


(a)

(b)

(c)

Supplementary table S1. MR acquisition parameters for the scanners used in this study

| Manufacturer | **Siemens** | | **GE** | | **Philips** | |
| --- | --- | --- | --- | --- | --- | --- |
| Field strength | 1.5T | 3T | 1.5T | 3T | 1.5T | 3T |
| Model | Avanto Fit | Prisma | Optima 450w | Discovery 750 | Ingenia | Ingenia |
| Native resolution  X*Y*Z | 1.1*1.1*1.1 mm | | | | | |
| Number of slices | 60 | 60 | 60 | 60 | 72 | 72 |
| Acquisition matrix | 256x256 | | | | | |
| Reconstruction matrix | 512x512 | | | | | |
| Echo time | 477 ms | 604 ms | 627 ms | 594 ms | 600 ms | 600 ms |
| Repetition time | Once per breath at expiration phase of breathing cycle, as detected by respiratory gating. Value depends on patient breathing frequency | | | | | |
| Echo train length | 200 | 200 | 120 | 120 | 180 | 160 |
| Parallel imaging | GRAPPA 2.0 | GRAPPA 2.0 | ARC  2.0 | ARC  2.0 | SENSE 2.0 | SENSE 2.0 |
| Distortion correction | ON | ON | 3D geometry correction | 3D geometry correction | Geometry correction ON | Geometry correction ON |
| Fat saturation | SPAIR | SPAIR | MRCP ChemSat: Fat | T2P ChemSat: Fat | SPIR | SPIR |

Supplementary table S2. Definitions of whole-tree metrics calculated by MRCP+

| **Metric** | **Definition** |
| --- | --- |
| Total number of ducts | The total number of modelled ducts |
| Biliary Tree Volume (mm^3^) | The volume of the total biliary tree |
| Gallbladder Volume (mm^3^) | The volume of the gall bladder |
| **Duct length metrics** |  |
| Duct length mean (mm) | Statistics on the mean length (mm) of the modelled ducts, measured along the centreline |
| Duct length sum (mm) | Total length of the centrelines in the modelled tree (mm) |
| Duct diameter < 3 mm (%) | The percentage of duct median diameters < 3 mm |
| Percentage of ducts with diameter [x, y] mm (%) | The percentage of duct median diameters in the interval [x, y] mm |
| Duct diameter ≥ 9 mm (%) | The percentage of duct median diameters ≥ 9 mm |
| **Strictures metrics** |  |
| Total number of strictures | The total number of strictures across all of the modelled ducts |
| Number of ducts with a stricture | The number of ducts containing one or more strictures |
| Stricture length sum (mm) | The total length (mm) of strictures |
| Stricture absolute severity sum (mm) | Total of following calculation across all modelled ducts: Minimum diameter – Closest maximum diameter |
| Stricture relative severity sum | Total of following calculation across all modelled ducts: (Absolute stricture severity / Closest maximum diameter) × 100 |
| Stricture score sum (mm) | Total of the stricture score (defined as Stricture length x× (Relative stricture severity / 100)) |
| **Dilatations metrics** |  |
| Total number of dilatations | The total number of dilatations across all of the modelled ducts |
| Number of ducts with a dilatation | The number of ducts containing one or more dilatations |
| Dilatation length sum (mm) | The total length (mm) of all dilatations |
| Dilatation diameter max (mm) | The maximum diameter (mm) of all dilatations |
| Dilatation absolute severity sum (mm) | Total of following calculation across all modelled ducts: Maximum diameter – Closest minimum diameter |
| Dilatation relative severity sum | Total of following calculation across all modelled ducts: (Absolute dilatation severity / Closest minimum diameter) x× 100 |
| Dilatation absolute severity max (mm) | Maximum of following calculation across all modelled ducts: Maximum diameter – Closest minimum diameter |
| Dilatation relative severity max | Maximum of following calculation across all modelled ducts: (Absolute dilatation severity / Closest minimum diameter) x× 100 |
| Dilatation score sum (mm) | Total of the dilatation score (defined as  dilatation length x× (relative severity / 100) |
| **Strictures and/or dilatations** |  |
| Number of ducts with a stricture or dilatation | The number of ducts with one or more strictures or dilatations |
| Number of ducts with a stricture and dilatation | The number of ducts with one or more strictures and one or more dilatations |
| Abnormal length sum (mm) | The total length (mm) of detected abnormalities (stricture length sum + dilatation length sum) |

Supplementary Table S3. Absolute stricture and dilatation metrics of the phantom calculated for each tube using the tube dimensions defined in the manufacturing specification.

| **Duct ID** | **Num. strictures** | **Stricture severity (mm)**  **Mean/Max/Sum** | | | **Stricture length (mm)**  **Mean/Max/sum** | | | **Num. dilatations** | **Dilatation severity (mm)**  **Mean/Max/sum** | | | **Dilatation length (mm)**  **Mean/Max/Sum** | | |
| --- | --- | --- | --- | --- | --- | --- | --- | --- | --- | --- | --- | --- | --- | --- |
| 1 | 1 | 1.9 | 1.9 | 1.9 | 7.4 | 7.4 | 7.4 | 1 | 1.7 | 1.7 | 1.7 | 19.2 | 19.2 | 19.2 |
| 2 | 1 | 2.9 | 2.9 | 2.9 | 10.9 | 10.9 | 10.9 | 1 | 1.8 | 1.8 | 1.8 | 26.5 | 26.5 | 26.5 |
| 3 | 1 | 4.2 | 4.2 | 4.2 | 9.5 | 9.5 | 9.5 | 1 | 4.8 | 4.8 | 4.8 | 26.1 | 26.1 | 26.1 |
| 4 | 1 | 4.4 | 4.4 | 4.4 | 8.2 | 8.2 | 8.2 | 1 | 3.2 | 3.2 | 3.2 | 19.2 | 19.2 | 19.2 |
| 5 | 1 | 7.2 | 7.2 | 7.2 | 6.8 | 6.8 | 6.8 | 2 | 6.2 | 6.6 | 12.4 | 15.0 | 15.7 | 30.0 |
| 6 | 1 | 4.0 | 4.0 | 4.0 | 17.3 | 17.3 | 17.3 | 1 | 3.0 | 3.0 | 3.0 | 18.8 | 18.8 | 18.8 |
| 7 | 1 | 7.0 | 7.0 | 7.0 | 18.0 | 18.0 | 18.0 | 2 | 3.0 | 3.0 | 6.0 | 12.0 | 12.5 | 24.0 |
| 8 | 1 | 7.9 | 7.9 | 7.9 | 30.4 | 30.4 | 30.4 | 2 | 5.0 | 5.0 | 9.9 | 14.3 | 14.6 | 28.6 |
| 9 | 1 | 3.7 | 3.7 | 3.7 | 2.8 | 2.8 | 2.8 | 1 | 3.6 | 3.6 | 3.6 | 2.7 | 2.7 | 2.7 |
| 10 | 1 | 5.8 | 5.8 | 5.8 | 7.4 | 7.4 | 7.4 | 2 | 5.8 | 5.8 | 11.6 | 6.9 | 7.1 | 13.9 |
| 11 | 2 | 4.3 | 4.3 | 8.6 | 12.1 | 12.3 | 24.3 | 3 | 3.7 | 4.3 | 11.2 | 8.2 | 9.7 | 24.6 |
| 12 | 2 | 5.9 | 5.9 | 11.8 | 9.2 | 9.2 | 18.3 | 3 | 4.6 | 5.9 | 13.9 | 5.0 | 6.5 | 15.0 |
| 13 | 3 | 5.0 | 6.3 | 15.1 | 12.4 | 12.7 | 37.2 | 4 | 5.0 | 6.3 | 19.9 | 8.0 | 8.6 | 31.9 |
| 14 | 4 | 4.2 | 4.6 | 16.7 | 5.5 | 5.8 | 21.9 | 5 | 4.4 | 8.4 | 22.0 | 5.4 | 6.7 | 27.1 |
| 15 | 2 | 3.0 | 3.0 | 6.0 | 16.7 | 16.7 | 33.3 | 3 | 2.3 | 3.0 | 7.0 | 9.1 | 11.0 | 27.4 |
| 16 | 1 | 1.0 | 1.0 | 1.0 | 3.2 | 3.2 | 3.2 | 1 | 1.0 | 1.0 | 1.0 | 2.5 | 2.5 | 2.5 |
| 17 | 2 | 3.9 | 3.9 | 7.8 | 6.0 | 6.3 | 12.0 | 3 | 2.6 | 3.9 | 7.9 | 4.6 | 6.2 | 13.8 |
| 18 | 2 | 6.0 | 8.0 | 12.0 | 11.8 | 14.8 | 23.7 | 2 | 6.0 | 8.0 | 12.0 | 11.6 | 14.8 | 23.2 |
| 19 | 3 | 6.7 | 8.0 | 20.0 | 8.5 | 9.8 | 25.5 | 3 | 6.7 | 8.0 | 20.0 | 8.4 | 9.8 | 25.2 |
| 20 | 3 | 6.9 | 7.0 | 20.7 | 9.1 | 9.2 | 27.2 | 4 | 5.4 | 7.0 | 21.7 | 6.3 | 7.7 | 25.1 |

Supplementary table S4. Inter-operator and Intra-operator of PSC specific metrics on the reference scanner (Siemens Prisma 3T). LoA – Limits of Agreement; RC – Reproducibility Coefficient.

| **Metric** | **Inter-operator** | | **Intra-operator** | |
| --- | --- | --- | --- | --- |
|  | **LoA** | **RC** | **LoA** | **RC** |
| Total number of ducts | [-24.2, 28.7] | 13.5 | [-16, 12.9] | 7.4 |
| Total number of dilatations | [-5.7, 4.6] | 2.6 | [-2.7, 2.3] | 1.3 |
| Dilatation length sum (mm) | [-5.7, 5.2] | 2.8 | [-5.2, 4.1] | 2.4 |
| Total number of strictures | [-3.9, 3.7] | 1.9 | [-4, 3.3] | 1.9 |
| Stricture length sum (mm) | [-43.3, 34.6] | 19.9 | [-27.5, 19.6] | 12 |
| Total number of ducts with a stricture or dilatation | [-5.5, 4.5] | 2.5 | [-2.8, 1.9] | 1.2 |
| Percentage of ducts with diameter 3-5 mm | [-0.2, 0.1] | 0.1 | [-0.1, 0.1] | 0 |

Supplementary Table S5: Accuracy of single duct metrics, calculated via Bland-Altman analysis comparing the measurements for each 3D printed phantom tube to those of the expected values. Bias results are shown, with the 95% limits of agreement shown in square brackets.

| **Metric** | **Siemens 1.5T** | **Siemens 3T** | **GE 1.5T** | **GE 3T** | **Philips 1.5T** | **Philips 3T** |
| --- | --- | --- | --- | --- | --- | --- |
| Duct length (mm) | 6.9  [4.7, 9.1] | 5.3  [2.4, 8.1] | 5.5  [2.3, 8.7] | 6.0  [3.3, 8.8] | 6.8  [2.1, 11.5] | 7.8  [5.0, 10.6] |
| Max. stricture length (mm) | 0.3  [-3.4, 4.0] | 0.1  [-3.9, 4.0] | 0.1  [-1.7,2.0] | 0.8  [-5.1, 6.6] | -0.2  [-1.6, 1.2] | -0.2  [-1.3, 0.9] |
| Mean stricture length (mm) | 0.3  [-3.3, 4.0] | 0.1  [-3.8, 4.0] | 0.1  [-1.7, 1.9] | 0.9  [-6.1, 7.9] | 0.0  [-1.7, 1.7] | -0.2  [-1.4, 1.0] |
| Stricture length sum (mm) | 0.4  [-3.3, 4.1] | 0.1  [-4.0, 4.2] | 0.3  [-1.9, 2.5] | 1.0  [-6.9,8.9] | -0.3  [-3.5, 2.9] | 0.0  [-1.9, 1.8] |
| Dilatation length sum (mm) | 0.9  [9.0, 10.9] | 1.7  [-8.3, 11.6] | 1.6  [-9.9, 13.1] | 0.5  [-13.8, 14.8] | 2.2  [-8.6, 12.9] | 0.6  [-16.1, 17.4] |
| Max dilatation diameter (mm) | 0.0  [-0.4, 0.4] | 0.0  [-1.1, 1.1] | -0.1  [-0.8, 0.6] | -0.3  [-1.2, 0.5] | 0.2  [-0.7, 1.1] | 0.3  [-0.6, 1.2] |
| Stricture absolute severity sum (mm) | 1.0  [-0.8, 2.8] | 1.1  [-1.0, 3.2] | 0.5  [-0.7, 1.7] | 0.5  [-2.0, 3.0] | 0.6  [-1.4, 2.5] | 0.9  [-0.7, 2.4] |
| Dilatation absolute severity sum (mm) | 1.0  [-1.0, 3.0] | 1.3  [-1.4, 3.9] | 0.7  [-0.7, 2.0] | 0.9  [-1.4, 3.2] | 0.7  [-1.3, 2.8] | 1.0  [-1.3, 3.3] |
| Max. dilatation absolute severity (mm) | 0.6  [-0.3, 1.5] | 0.6  [-0.5, 1.6] | 0.4  [-0.4, 1.2] | 0.6  [-0.5, 1.6] | 0.3  [-0.6, 1.3] | 0.5  [-0.7, 1.6] |
| Stricture relative severity sum (%)* | 0.1  [-0.1, 0.4] | 0.2  [-0.1, 0.5] | 0.1  [-0.1, 0.2] | 0.1  [-0.3, 0.4] | 0.0  [-0.3, 0.4] | 0.1  [-0.1, 0.3] |
| Dilatation relative severity sum (%)* | 0.5  [-0.6, 1.6] | 0.5  [0.4, 1.5] | 0.4  [-0.5, 1.3] | 0.5  [-0.4, 1.4] | 0.2  [0.4, 0.9] | 0.3  [-0.5, 1.1] |
| Max. dilatation relative severity (%)* | 0.3  [-0.2, 0.9] | 0.3  [-0.3, 0.9] | 0.3  [-0.3, 0.8] | 0.3  [-0.2, 0.8] | 0.1  [-0.3, 0.6] | 0.2  [-0.4, 0.8] |
| Stricture score sum (mm) | 1.2  [-0.9, 3.3] | 1.3  [-1.5, 4.1] | 0.9  [-1.1, 2.8] | 1.5  [-4.8, 7.8] | 0.6  [-2.3, 3.6] | 0.9  [-1.6, 3.4] |
| Dilatation score sum (mm) | 4.8  [-2.8, 12.4] | 4.6  [-3.5, 12.7] | 3.9  [-3.2, 11.0] | 4.9  [-3.0, 12.7] | 2.7  [-3.6, 9.0] | 2.9  [-8.8, 14.8] |
| Number of dilatations | 0.1  [-0.4-0.5] | 0.1  [-0.4, 0.5] | 0.1  [-0.4, 0.5] | 0.1  [-0.9, 1.0] | 0.1  [-0.5, 0.7] | 0.0  [-1.0, 1.0] |
| Number of strictures | 0.1  [-0.4,0.5] | 0.2  [-0.6, 1.0] | 0.0  [-0.0, 0.0] | -0.1  [-0.6, 0.5] | -0.1  [-0.5, 0.4] | 0.0  [-0.0, 0.0] |

**note metrics whose units are percentage points are shown as fractions here – to convert to percentage points they need to be multiplied by 100.*

Supplementary table S6. Reference ranges for all MRCP+ metrics.

| **Metric** | **Mean** | **Median** | **SD** | **IQR** | **Reference Range - Lower Bound** | **Reference Range - Upper Bound** |
| --- | --- | --- | --- | --- | --- | --- |
| Total number of ducts | 21.4 | 20 | 9.3 | 10.5 | 0 | 38 |
| Duct length sum (mm) | 547.6 | 490.2 | 260.7 | 278.5 | 0 | 1112.9 |
| Total number of dilatations | 3.5 | 3 | 2.4 | 2.5 | 0 | 8 |
| Dilatation length sum (mm) | 34.9 | 33.8 | 23.9 | 27.1 | 0 | 75.7 |
| Total number of strictures | 2.3 | 2 | 1.8 | 2 | 0 | 6 |
| Stricture length sum (mm) | 22.4 | 20.7 | 18.9 | 18.6 | 0 | 59.6 |
| Stricture absolute severity sum (mm) | 3.2 | 3 | 2.5 | 2.9 | 0 | 7 |
| Total number of ducts with a stricture or dilatation | 4.1 | 4 | 2.3 | 3.5 | 1 | 9 |
| Duct length mean (mm) | 25.7 | 25.4 | 4.7 | 5.4 | 17.7 | 35.5 |
| Total number of ducts with a stricture and dilatation | 1.2 | 1 | 1.2 | 2 | 0 | 3 |
| Number of ducts with a stricture | 2.2 | 2 | 1.7 | 2 | 0 | 5 |
| Number of ducts with a dilatation | 3.1 | 3 | 1.9 | 2 | 0 | 6 |
| Dilatation score sum (mm) | 21.1 | 18 | 14.7 | 16.5 | 0 | 45.6 |
| Dilatation absolute severity max (mm) | 2.1 | 2 | 0.9 | 1.1 | 0.3 | 3.9 |
| Dilatation absolute severity sum (mm) | 5.5 | 4.5 | 3.6 | 3.5 | 0 | 13.2 |
| Dilatation relative severity sum | 2.2 | 1.8 | 1.5 | 1.5 | 0 | 5.8 |
| Dilatation relative severity max | 0.8 | 0.8 | 0.4 | 0.3 | 0 | 2 |
| Dilatation diameter max (mm) | 5.7 | 5.8 | 1.3 | 1.6 | 3.1 | 8.4 |
| Stricture score sum (mm) | 9.1 | 8 | 8 | 8 | 0 | 23 |
| Stricture relative severity sum | 1 | 0.8 | 0.8 | 0.8 | 0 | 2.4 |
| Abnormal length sum (mm) | 57.3 | 55.4 | 34.5 | 39.1 | 0 | 119.8 |
| Biliary tree volume | 4.3 | 3.8 | 2 | 1.9 | 0 | 8 |
| Gallbladder volume | 12.7 | 11.4 | 9 | 11 | 0 | 26.3 |
| Percentage of ducts with diameter <3 mm | 80 | 80 | 10 | 10 | 57 | 100 |
| Percentage of ducts with diameter 3-5 mm | 21 | 20 | 9 | 11 | 0 | 38 |
| Percentage of ducts with diameter 5-9 mm | 0 | 0 | 0 | 0 | 0 | 8 |
| Percentage of ducts with diameter >9 mm | 0 | 0 | 0 | 0 | 0 | 0 |

Supplementary Table S7: Repeatability Coefficients of all Whole Tree metrics

| **Metric** | **GE 1.5T** | **GE 3T** | **Siemens 1.5T** | **Siemens 3T** | **Philips 1.5T** | **Philips 3T** |
| --- | --- | --- | --- | --- | --- | --- |
| Total number of ducts | 14.8 | 12.2 | 23.5 | 10.3 | 13.4 | 10.3 |
| Duct length sum (mm) | 355 | 311.2 | 467.4 | 228.5 | 354.3 | 242.8 |
| Total number of dilatations | 5.9 | 4.8 | 5.8 | 4.4 | 4.6 | 2.7 |
| Dilatation length sum (mm) | 51 | 31 | 56.2 | 41.5 | 46 | 43.4 |
| Total number of strictures | 3.8 | 3.9 | 5.3 | 4.6 | 4.4 | 3.1 |
| Stricture length sum (mm) | 35.8 | 25.2 | 47.4 | 49.6 | 49.1 | 22.7 |
| Stricture absolute severity sum (mm) | 5.5 | 6.7 | 6.9 | 6.8 | 6 | 4.2 |
| Total number of ducts with a stricture or dilatation | 5.5 | 5 | 5.8 | 4.7 | 3.8 | 2.2 |
| Duct length mean (mm) | 6.4 | 6.1 | 7.7 | 6.7 | 4.9 | 8.3 |
| Total number of ducts with a stricture and dilatation | 2.9 | 2.7 | 3 | 3.2 | 2.3 | 1.4 |
| Number of ducts with a stricture | 3.3 | 2.4 | 4.6 | 4.3 | 2.9 | 3.1 |
| Number of ducts with a dilatation | 5.3 | 4.4 | 4.9 | 3.6 | 3.6 | 2.7 |
| Dilatation score sum (mm) | 30.9 | 28.9 | 38.9 | 28.8 | 30.6 | 29.2 |
| Dilatation absolute severity max (mm) | 1.6 | 1.8 | 1.8 | 1.5 | 2.1 | 1.4 |
| Dilatation absolute severity sum (mm) | 6.9 | 6.7 | 8.4 | 6.7 | 7.7 | 4 |
| Dilatation relative severity sum | 3.6 | 3.7 | 3.5 | 3.1 | 3.3 | 1.9 |
| Dilatation relative severity max | 0.8 | 0.7 | 0.7 | 0.8 | 0.8 | 0.9 |
| Dilatation diameter max (mm) | 1.8 | 2.9 | 1.9 | 1.9 | 2.3 | 3.4 |
| Stricture score sum (mm) | 15.4 | 10.8 | 18.1 | 20.1 | 20.4 | 10.1 |
| Stricture relative severity sum | 1.7 | 1.8 | 2 | 2.1 | 1.8 | 1.4 |
| Abnormal length sum (mm) | 73.8 | 52.5 | 78.4 | 65.1 | 72.9 | 44.5 |
| Biliary tree volume | 1.8 | 1.7 | 2.9 | 1.9 | 2.2 | 1.4 |
| Gallbladder volume | 5.9 | 11.8 | 19 | 9.9 | 8 | 9.4 |
| Percentage of ducts with diameter <3 mm | 0.3 | 0.1 | 0.3 | 0.1 | 0.2 | 0.3 |
| Percentage of ducts with diameter 3-5 mm | 0.3 | 0.1 | 0.3 | 0.2 | 0.2 | 0.2 |
| Percentage of ducts with diameter 5-9 mm | 0.1 | 0.1 | 0 | 0.1 | 0.1 | 0.2 |
| Percentage of ducts with diameter >9 mm | 0 | 0 | 0 | 0 | 0 | 0 |

*note metrics whose units are percentage points are shown as fractions here – to convert to percentage points they need to be multiplied by 100.

Supplementary Table S8: Reproducibility Coefficients of all Whole Tree metrics

| **Metric** | **GE 1.5T** | **GE 3T** | **Siemens 1.5T** | **Philips 1.5T** | **Philips 3T** |
| --- | --- | --- | --- | --- | --- |
| Total number of ducts | 20.4 | 13.4 | 25.4 | 14.7 | 12.4 |
| Duct length sum (mm) | 497.8 | 330.9 | 384.8 | 340.6 | 342.2 |
| Total number of dilatations | 6.7 | 4.9 | 6.4 | 7.9 | 6.6 |
| Dilatation length sum (mm) | 63.2 | 45.2 | 62.1 | 57.5 | 60.1 |
| Total number of strictures | 4.1 | 4.3 | 6.5 | 3.5 | 3.3 |
| Stricture length sum (mm) | 51.6 | 37 | 51.7 | 26.5 | 37.4 |
| Stricture absolute severity sum (mm) | 6 | 7.5 | 9.8 | 4.6 | 4.7 |
| Total number of ducts with a stricture or dilatation | 4.5 | 4.4 | 6.8 | 6 | 5.6 |
| Duct length mean (mm) | 9.7 | 9.3 | 7.4 | 7.7 | 8.6 |
| Total number of ducts with a stricture and dilatation | 3.2 | 2.4 | 3.8 | 2.6 | 2 |
| Number of ducts with a stricture | 3.9 | 3.8 | 5.8 | 2.8 | 3.3 |
| Number of ducts with a dilatation | 4.6 | 3.2 | 5.5 | 6.2 | 5.4 |
| Dilatation score sum (mm) | 48.8 | 44.4 | 40.4 | 37.7 | 31.8 |
| Dilatation absolute severity max (mm) | 1.4 | 2.4 | 2 | 2.2 | 2.3 |
| Dilatation absolute severity sum (mm) | 9.4 | 9.1 | 8.8 | 11.8 | 8.7 |
| Dilatation relative severity sum | 4.5 | 3.9 | 4.1 | 5.1 | 3.8 |
| Dilatation relative severity max | 0.5 | 0.9 | 0.8 | 1.4 | 1.2 |
| Dilatation diameter max (mm) | 2.1 | 2.6 | 2.6 | 3 | 3.7 |
| Stricture score sum (mm) | 20.5 | 16.5 | 21.9 | 11.5 | 15.6 |
| Stricture relative severity sum | 1.6 | 1.9 | 2.9 | 1.5 | 1.3 |
| Abnormal length sum (mm) | 93.5 | 64.8 | 86.5 | 64.2 | 63.6 |
| Biliary tree volume | 2.7 | 2.5 | 2.7 | 2.5 | 2.3 |
| Gallbladder volume | 16 | 11.6 | 20.6 | 16.9 | 14.1 |
| Percentage of ducts with diameter <3 mm | 0.3 | 0.2 | 0.3 | 0.3 | 0.3 |
| Percentage of ducts with diameter 3-5 mm | 0.3 | 0.2 | 0.3 | 0.3 | 0.3 |
| Percentage of ducts with diameter 5-9 mm | 0 | 0 | 0.1 | 0.1 | 0.2 |
| Percentage of ducts with diameter >9 mm | 0 | 0 | 0 | 0 | 0 |

*note metrics whose units are percentage points are shown as fractions here – to convert to percentage points they need to be multiplied by 100.
